# Comparative Electroanalytical Studies of Graphite Flake and Multilayer Graphene Paste Electrodes

**DOI:** 10.3390/s20061684

**Published:** 2020-03-18

**Authors:** Natalia Festinger, Kamila Morawska, Vladimir Ivanovski, Magdalena Ziąbka, Katarzyna Jedlińska, Witold Ciesielski, Sylwia Smarzewska

**Affiliations:** 1 Department of Inorganic and Analytical Chemistry, Faculty of Chemistry, University of Lodz, 91-403 Lodz, Poland; 2 Institute of Chemistry, Faculty of Natural Sciences and Mathematics, University of Ss. Cyril and Methodius in Skopje, 1000 Skopje, Macedonia; 3 Faculty of Materials Science and Ceramics, AGH University of Science and Technology, 30-059 Cracow, Poland

**Keywords:** graphite flake, multilayer graphene, carbon paste electrode, scanning electron microscopy, voltammetry

## Abstract

In this paper, the fabrication, surface characterisation and electrochemical properties of graphite flake (GFPE) and multilayer graphene (MLGPE) paste electrodes are described. The Raman investigations and scanning electron microscopy were used to analyze and compare structure of both carbon materials. The electroanalytical performance of both electrodes was examined and compared on the basis of the square-wave and cyclic voltammetric behavior of acetaminophen and model redox systems. Results of those studies revealed that GFPE has a larger electroactive surface area and better conductive properties, whilst MLGPE demonstrate better analytical characteristic in case of acetaminophen (AC) determination. AC determination was developed using square wave voltammetry (SWV) and square wave stripping voltammetry (SWSV). For both working electrodes, the process of accumulation enabled us to obtain an extended linear range and to lower the detection limit. In pharmaceutical formulations, AC was determined with good recovery.

## 1. Introduction

Carbon occurs in many allotropic forms. Amorphous carbon, diamond, and graphite were the only known forms of carbon [1], until fullerenes were discovered in 1985 [2]. It initiated an increasing interest in carbon forms, leading to the discovery of carbon nanotubes [3,4] and graphene [5]. The properties of diamond, graphite, or graphene show that every form of the carbon element is different and each has its own unique characteristic [1].

Paste electrodes based on a mixture of graphite powder and a pasting liquid have been very popular since their discovery [6,7]. The specific properties of the carbon paste depend on the type and quality of the carbon powder. Many known forms of carbon can be used for the production of paste electrodes, for example, synthetic graphite, glassy carbon or multi-walled carbon nanotubes [8,9,10]. Electrodes composed of carbon paste are still widely used in electrochemical studies because of their wide potential window, high sensitivity, low background current, and low cost [11]. Many biologically active compounds, e.g., pesticides [12,13], pharmaceuticals [14,15,16], and ions [17,18,19], can be determined using CPEs. 

Graphite, the most widespread form of carbon, is also the most commonly used material for the production of paste electrodes. Graphite flake (GF) is a type of graphite occurring in nature. GF shows anisotropic thermal conductivity, with high conductivity in the horizontal direction and low thermal conductivity in the vertical direction [20]. Graphite flakes have found application in electrochemistry as, for instance, a promising anode material for lithium ion batteries [21]. GFs have unique features, including excellent strength and high fracture toughness [22]. Furthermore, the layered graphite structure and weak forces between the layers lead to the possibility of easily refreshing the surface of a paste electrode made of graphite flakes. These properties make graphite flake a promising electrode material [23].

Recently, graphene is considered the material of the "rising star" and has received attention due to its unique properties. This form of carbon is considered as a prototype of two-dimensional carbon system and all other dimensionalities [5,24,25]. Graphene can be wrapped into fullerenes (0D), rolled into carbon nanotubes (1D), or used to create graphite (3D) [24]. The development of knowledge related to graphene led to the creation of graphene-based materials. Multilayer graphene (MLG) is a 2D nanomaterial composed of stacked monolayers of graphene. Importantly, both MLG and GNP (graphene nanoplatelets) are composed of graphene layers arranged on top of each other. However, they differ in the number of layers and properties. The number of graphene layers in GNP is greater than 10, while that in MLG varies from 11 to 21 [26]. Experiments show that for model redox systems there is no strong correlation between the flake thickness and the electron transfer rate [27]. However, a higher electron transfer was achieved for flakes consisting of less than 20 graphene layers [27]. The properties of MLG are more similar to those of graphene than to those of graphite [26], and thus make MLG a promising material for the fabrication of carbon paste electrodes. 

Acetaminophen (AC) is very commonly used in many diseases and pain treatment. Because of its antipyretic and analgesic properties, AC is one of the most frequently prescribed painkillers in the world [28]. It is worth noting that too high doses or long-term use may cause undesirable effects in the body such as severe hepatotoxicity and nephrotoxicity because of the accumulation of toxic metabolites. Therefore, it is very important to precisely define and control the quality [29]. A number of analytical techniques were used for AC determination, such as UV spectrophotometry [30], reverse-phase high-performance liquid chromatography [31], high-performance thin-layer chromatography [32], liquid chromatography-tandem mass spectrometry [33], thin-layer chromatography [34], and capillary electrophoresis [35]. Electrochemical techniques, which are characterized by the cheapness, ease and speed of the analysis, were also used for AC determination using many types of carbon electrodes including carbon paste electrodes [36,37,38,39,40], glassy carbon electrode [41,42] or screen-printed carbon electrodes [43,44]. Acetaminophen is also often used in electrochemical analysis to compare analytical performance of the developed sensors [45,46,47,48] as its electrochemical properties have been thoroughly examined. Therefore, in this research, AC was chosen to compare and describe the analytical utility of two working paste electrodes based on selected carbon materials.

The aim of the present research was to compare the suitability of graphite flakes and multilayer graphene for the production of paste electrodes. Comparative tests of the obtained paste electrodes were conducted using the acetaminophen as a model compound. Properties of carbon paste electrodes were studied using voltammetric techniques (CV, SWV and SWSV), scanning electron microscopy and Raman spectroscopy. Examination of the electrochemical properties of GFPE and MLGPE may be helpful for researchers in the selection of electrode materials for future studies, as properly chosen electrode materials allow us to achieve better analytical parameters (e.g., sensitivity, limit of detection) of the method. There are many articles in the literature comparing different types of carbon material for the production of paste electrodes (e.g., graphene, graphite, carbon black, multi-walled carbon nanotubes, nanocarbon, glassy carbon) [8,49,50]. However, to the best of our knowledge, the comparison of the electrochemical and electroanalytical properties of GFPE and MLGPE has not been investigated so far. In the presented paper, the full characterization is displayed and discussed thoroughly. Suggestions on the electrochemical usefulness of both materials is pointed out.

## 2. Materials and Methods

### 2.1. Instrumentation and Working Electrode Preparation

Electrochemical studies were carried out using μAutolab Type III (Metrohm Autolab B.V., Utrecht, Netherlands) controlled by the GPES program (version 4.9) and a M164 electrode stand (mtm-anko, Cracow, Poland) with a three-electrode system consisting of Ag/AgCl (3.0 mol L^−1^ KCl) as a reference electrode, Pt wire as an auxiliary electrode, and a carbon paste electrode (GFPE or MLGPE) as a working electrode. To make the CPEs GFPE and MLGPE, 300 μL of paraffin oil was mixed with 1.0 g of graphite flakes and multilayer graphene, respectively. Then, the mixtures were hand-mixed in a mortar for 20 minutes until a homogeneous paste was obtained. Next, the paste was packed into a Teflon tube with inner diameter 3 mm (geometric area of electrode was equal to 7.069 mm^2^) and a metal contact. Before each experiment, the electrode surface was refreshed by squeezing out a small portion of paste and polishing it using a wet filter paper until a smooth surface was obtained. An appropriately prepared electrode shows a stable electrochemical response. Ultra-pure water was obtained from DL3–90 deionizer (Labopol-Polwater, Cracow, Poland). All pH measurements were conducted with a pH meter (Elmetron CP-315, Elmetron Sp.j., Zabrze, Poland) and a conjugated glass membrane electrode. The Raman spectra were recorded using the 633 nm He-Ne laser line on a Horiba Jobin Yvon LabRAM 300. The laser power employed on the samples was 3.29 mW by using 120 or 150 s exposition per diffraction window. In all the measurements, a 50x Olympus lens (long range), a hole of 500 μm, a slit of 100 μm, and a diffraction grid with 1800 grooves/mm were employed. To perform a detailed study of the microstructure of the electrode surface, a scanning electron microscope (Nova NanoSEM 200 FEI, Netherlands) was used. The SEM observations were carried out at an 18 kV accelerating voltage and in low vacuum conditions (60 Pa). 

### 2.2. Materials and Solutions

Acetaminophen was purchased from Sigma-Aldrich (Saint Louis, Missouri, USA), graphite flakes (median 7–10 microns, purity 99%) were purchased from Alfa Aesar (Kandel, Germany), and multilayer graphene was obtained from Graphene Supermarket (Reading, Massachusetts, USA). Paraffin oil and other chemicals were purchased from Avantor (Gliwice, Poland). A 1.0 mmol L^−1^ stock solution of acetaminophen was prepared weekly by dissolving 1.51 mg of the compound in 10 mL of distilled water. Lower concentrations were obtained by dilution. Acetaminophen solution was stored in a cold and dark place. Britton–Robinson buffer components (acetic acid, boric acid, phosphoric acid and sodium hydroxide), citrate buffer components (sodium citrate, hydrochloric acid), and citrate-phosphate buffer components (disodium phosphate, citric acid) were purchased from Avantor (Gliwice, Poland). The drugs containing AC, i.e., Apap (AC 500 mg; US Pharmacia, Wroclaw, Poland), Apap Extra (AC 500 mg + caffeine 65 mg; US Pharmacia), Panadol (AC 500 mg; GlaxoSmithKline, London, Great Britain), and Paracetamol Polfa Łódź (AC 500 mg, Polfa Łódź, Lodz, Poland) were purchased from a local drugstore. The drug solutions containing AC in the concentration of 1.0 mmol L^−1^ were prepared weekly as follows: appropriate amounts of crushed drugs (2.51 mg Apap, 2.93 mg Apap Extra, 2.69 mg Panadol, and 3.63 mg Paracetamol Polfa Łódź) were dissolved in 15 mL of distilled water. Drug solutions were stored in a cold and dark place. In all experiments, voltammograms were recorded under the same conditions as those for pure acetaminophen. The drug solutions were analyzed using the standard addition method. To obtain the final concentrations of AC in the range of the calibration curve, the drug solutions were suitably diluted with the supporting electrolyte. Recoveries were calculated after three replicate experiments.

### 2.3. Voltammetric Procedure

The main procedure used to obtain voltammograms was as follows: an appropriate amount of compound was added to the voltammetric cell containing the supporting electrolyte. For SWSV, the pre-concentration step consisted of solution stirring at the chosen accumulation potential for the selected accumulation time. After the pre-concentration step, the stirrer was stopped, and the voltammogram was recorded in the potential window. All measurements were carried out at room temperature.

### 2.4. Statistics

The linear range of the acetaminophen peak current (*I*_p_, μA) versus its concentration (*C*, mol L^−1^) was plotted and described with the linear regression equation y = ax + b. Using the calibration curve, the limit of detection (LOD) and limit of quantification (LOQ) were calculated to estimate the sensitivity of the method. LOD and LOQ were evaluated by the following equations: 3·SD/a (for LOD) and 10·SD/a (for LOQ), where SD is standard deviation of intercept and a is slope of the calibration curve [51]. The recoveries were calculated using the formula: recovery [%] = 100 × (found /added). The precision was also calculated for each concentrations in the linear range to verify the accuracy of the method. For various concentrations, the coefficient of variation was calculated with the formula: CV = (SD × 100% / C_av_), where C_av_ is average acetaminophen concentration calculated from the linear regression equation and SD is standard deviation between those values. 

## 3. Results and Discussion

### 3.1. Electrochemical Properties of Working Electrodes

Cyclic voltammetry (CV) is a valuable technique used to compare the electrochemical properties of various electrodes. The voltammetric performance of two types of working electrodes, namely GFPE and MLGPE, was compared using K_4_[Fe(CN)_6_]/K_3_[Fe(CN)_6_] as a model redox system. 

**Figure 1:**
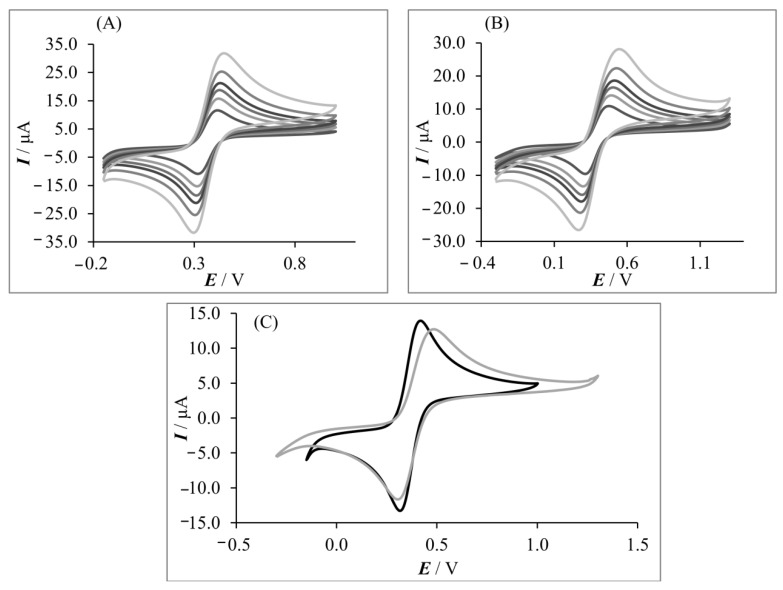
(**A**,**B**) Cyclic voltammograms for 1.0 mmol L^−1^ K_4_[Fe(CN)_6_]/K_3_[Fe(CN)_6_] (1:1) in Britton–Robinson buffer pH 5.0 at different scan rates of: 50, 100, 150, 200, 300, 500 mV·s^−1^ on (A) GFPE and (B) MLGPE; (**C**) Cyclic voltammograms for 1.0 mmol L^−1^ K_4_[Fe(CN)_6_]/K_3_[Fe(CN)_6_] (1:1) in Britton–Robinson buffer pH 5.0 as the supporting electrolyte on GFPE (black line) and MLGPE (gray line) with a scan rate of 50 mV·s^−1^.

Cyclic voltammograms of 1.0 mmol L^−1^ K_4_[Fe(CN)_6_]/K_3_[Fe(CN)_6_] were recorded on both types of electrodes. Sample cyclic voltammograms of K_4_[Fe(CN)_6_]/K_3_[Fe(CN)_6_] system are shown in Figure 1A–C. As can be seen from the figure, a well-defined redox couple was observed at both electrodes. Anodic-to-cathodic peak current ratios (*I_pa_*/*I_pc_*) for 50 mV s^−1^ were determined and were equal to 0.994 and 1.015 for GFPE and MLGPE, respectively.

The peak-to-peak separation (Δ*E_p_*) of cathodic and anodic signals of model redox system for 50 mV s^−1^ were 97 mV and 181 mV for GFPE and MLGPE, respectively. For every tested scan rate, Δ*E_p_* was lower on GFPE.

Moreover, to evaluate the rate of the electron transfer on GFPE and MLGPE electrodes, the relative peak separations (χ^0^) were calculated based on the following equation: χ^0^ = (*E_pa_* − *E_pc_*)/0.059 [52]. It was found that the GFPE showed a smaller value of χ^0^ (1.644) than MLGPE (3.068). This result indicates faster kinetics of [Fe(CN)_6_]^3−/4−^ redox couple on GFPE.

Taking into account differences in electron transfer kinetics and the fact that both working electrodes had the same geometric area, the electroactive surface for both electrodes was established. 

The electroactive surface area of working electrodes was calculated using dependence between peak current and square root of scan rate (*I*_p_ = *f*(*v* ^1/2^) in scan rate range of 50–500 mV·s^−1^) obtained on the basis of cyclic voltammetry measurements of ferrocyanide/ferricyanide redox couple. The electroactive surface area was calculated from Randles–Sevčik equation: *I*_p_ = 2.69 × 10^5^n^3/2^AC*D^1/2^*v*^1/2^, where *I*_p_ is peak current, n is number of electrons (n = 1), A is electroactive surface area of the electrode, C* is the concentration of potassium ferrocyanide (1.0 mmol L^−1^), D is the diffusion coefficient (7.60 × 10^−6^ cm^2^ s^−1^ [53]), and *v* is the scan rate. The electroactive surface area was equal to 5.14 ± 0.07 mm^2^ (n = 3) and 4.40 ± 0.06 mm^2^ (n = 3) for GFPE and MLGPE, respectively. The roughness factor (RF) of the electrodes was calculated by dividing the electrochemically active area by the geometric area and was equal to 0.741 (for GFPE) and 0.622 (for MLGPE). The RF could be attributed to the morphology of the working paste electrode. Thus, GFPE has a larger electroactive surface area and better conductive properties than MLGPE.

### 3.2. SEM Characterization of GFPE and MLGPE

**Figure 2:**
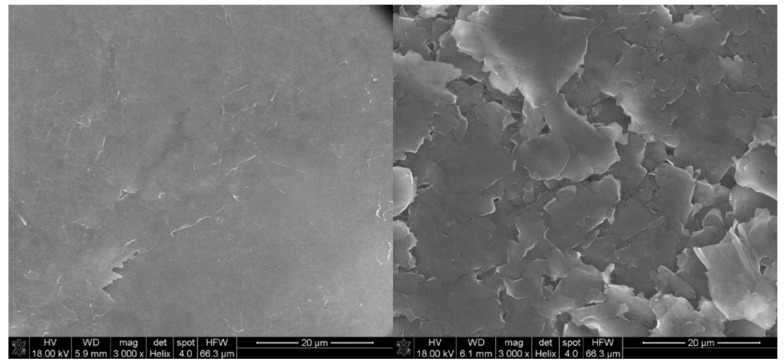
SEM images of multilayer graphene paste electrode (left) and graphite flake electrode (right).

Furthermore, to obtain information about the physicochemical properties of the GFPE and MLGPE electrodes, the topography and morphology characterizations of both types of CPEs were performed using scanning electron microscopy (SEM). As shown in Figure 2, the obtained images clearly indicate morphological differences between MLGPE and GFPE.

The SEM micrographs reveal that the surface of MLGPE is uniformly covered by the tightly distributed flakes. An extremely smooth surface morphology is observed, and the pore formation between flakes is poorly visible. In contrast, the surface of GFPE exhibits an extremely thin film and much more wrinkled structure than the previous electrode. The SEM image clearly depicts that this electrode shows an original crumbled multilayer structure with loose flaky design and rippled edged, paper-like sheet structures. A few layers of an expanded structure were uniformly distributed over the entire surface. A flake-like structure suggests an increase in the exposed surface area. 

### 3.3. Raman Spectra of Working Electrodes

**Figure 3:**
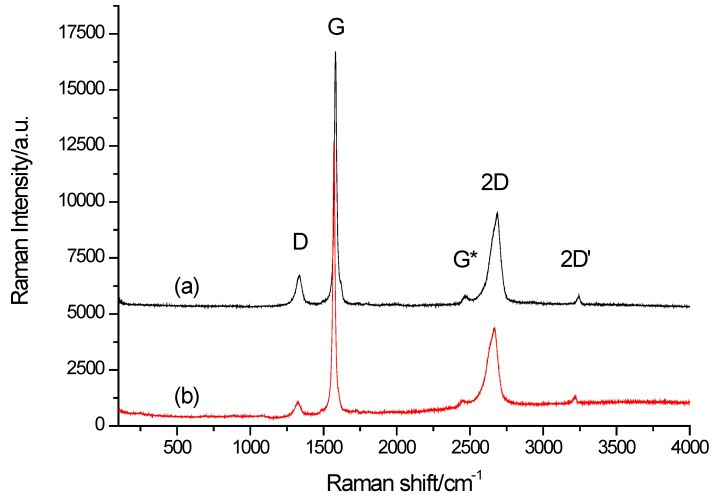
Raman spectra of the samples: (**a**) MLG, (**b**) GFs, recorded using the 623.8 nm He-Ne laser line. The characteristic D (≈ 1360 cm^−1^), G (≈ 1560 cm^−1^), 2D (≈ 2700 cm^−1^), and 2D’ (≈ 3250 cm^−1^) bands for polycarbonate specimens are marked.

The Raman spectra of the investigated samples are given in Figure 3. The Raman spectra of the two samples are consistent with the one defined in the literature for graphene and graphite [54,55,56]. The assignment is made according to data given in [56].

Although at the first glance, the spectra of MLG and graphite appear identical, there is a blue shift for the MLG bands in respect to the corresponding bands in GFs (D—9 cm^−1^, G—10 cm^−1^, 2D—17 cm^−1^ and 2D’—24 cm^−1^). It was confirmed that this shift was not due to pure non calibration of the instrument. As indicated by Ferrari [55], a pronounced shift is particularly expected for the 2D band with an increasing number of the graphene layers. Thus, it can be concluded that these materials are different if the ratio between the integrated intensities of the D and G bands *I*_D_/*I*_G_ for the two materials is calculated. This ratio gives an insight into the disordered structure, and the larger the ratio, the larger the disorder. The ratios for MLG and graphite are 0.286 and 0.102, respectively. The MLG sample is more disordered than the graphite, which is probably due to the larger amount of inclusions into the structure of the layers of MLG. In addition to this parameter, it is possible to use the relationship between the lateral dimensions of the crystallites in the sample and the reciprocal value of *L*_a_’ [57]. Countering the dispersion of the D band (for the used wavelength of 632.8 nm), this relationship is given as *L*_a_/nm = 38.484 (*I*_D_/*I*_G_) ^−1^. From the obtained results, a much higher value for the *L*_a_ of the crystallites is observed for the graphite (377.29 nm) than for the MLG sample (134.56 nm). The Raman spectra indicate that the structure of both examined specimens is different, and hence, one can expect different electrochemical properties. 

### 3.4. Electrochemical Behavior of Acetaminophen

**Figure 4:**
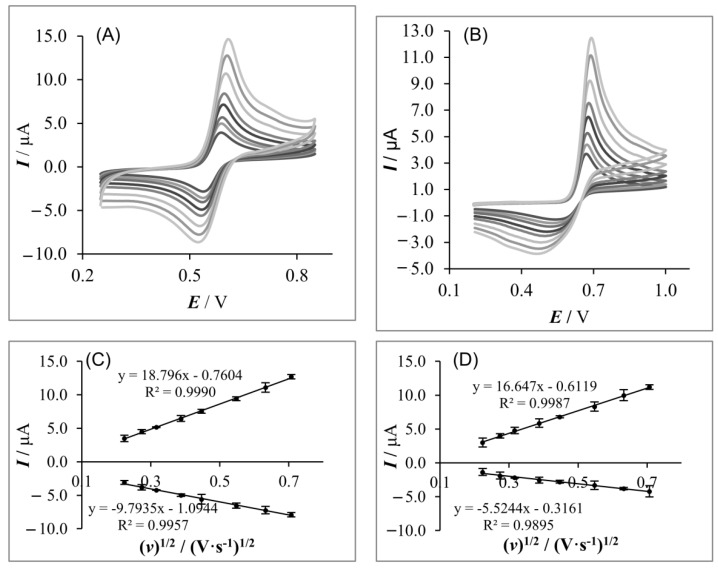
Cyclic voltammograms of 0.1 mmol L^−1^ AC on GFPE (**A**) and MLGPE (**B**) at different scan rates of 50, 75, 100, 150, 200, and 300 mV·s^−1^ in Britton–Robinson buffer at pH 5.0. (**C**) The dependence of AC peak current on square root of scan rate for GFPE (n = 3) and (**D**) The dependence of AC peak current on square root of scan rate for MLGPE (n = 3).

First, the electrochemical behavior of acetaminophen on both working electrodes was investigated by CV. The measurements were performed in the Britton–Robinson buffer (pH 5.0) in the presence of acetaminophen (0.1 mmol L^−1^). As shown in Figure 4, a pair of well-defined peaks was observed at both working electrodes. Figure 4 demonstrates differences in the electron transfer during the AC oxidation process at both electrodes—the reversible and quasi-reversible behavior of acetaminophen on GFPE and MLGPE, respectively. Taking into account signal morphology, it can be assumed that rate of electron transfer is lower for MLGPE. The oxidation peak of AC was observed at about 0.65 V at the GFPE and at about 0.70 V at MLGPE. The reduction peak of AC at MLGPE was broad and weak while at the GFPE, the reduction peak was more sharpen, which showed that the graphite flakes paste electrode act as an more effective promoter to enhance the kinetics of the electrochemical process. In terms of peak-to-peak separation, a lower ΔE indicates faster electron transfer kinetics and this parameter can be useful in order to optimize analytical systems. The transfer coefficient (α) was calculated according to Tafel plot [58] of AC and α was equal to 0.40 and 0.52 for MLGPE and GFPE, respectively. Calculated α explains the asymmetrical AC signal morphology on MLGPE. According to [59] for α < 0.5, the cathodic signal is more rounded than the anodic signal. This widening of obtained cathodic signal also reduces the peak height. Then, the heterogeneous electron transfer rate constant (k^0^) was calculated using the Nicholson method [59]. The heterogeneous electron transfer rate constant was equal to 0.00421 cm·s^−1^ and 0.364 cm·s^−1^ on MLGPE and GFPE, respectively. It is therefore confirmed that the kinetics of electron transfer on MLGPE is much slower than on GFPE. Calculated value of k^0^ confirms the assumption of the quasi-reversible and reversible behavior of acetaminophen on MLGPE and GFPE, respectively [60].

The scan rate effect on anodic and cathodic AC signals was then investigated. For scan rates in the range 50–500 mV·s^−1^, the peak currents increased linearly with the increasing square root of scan rates; this finding indicates that the electrochemical reaction of acetaminophen on GFPE and MLGPE is diffusion-controlled. Additionally, the plot of log *I*_p_ vs. log v showed a straight line with the linear regression equations as log *I*_p_ [A] = 0.55 × log v [V·s^−1^] − 6.38 (R^2^ = 0.9993) and log *I*_p_ [A] = 0.56 × log v [V·s^−1^] − 6.45 (R^2^ = 0.9942) for GFPE and MLGPE, respectively. For pure diffusive process, the slope of plots log *I*_p_ vs. log v should be equal to 0.5, and for the pure adsorptive process, it is equal to 1.0. According to this assumption, the oxidation process of AC is mainly controlled by diffusion; however, a slight influence of adsorption is visible. The nature of the electrode process is confirmed by the available literature report [30]. 

### 3.5. Optimization of Electrochemical Measurement Conditions

**Figure 5:**
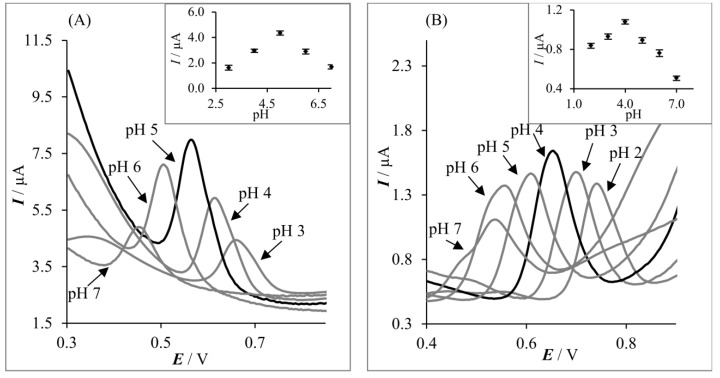
SW voltammograms of 5.0 μmol L^−1^ acetaminophen recorded in BR buffers for (**A**) GFPE and (**B**) MLGPE. The inset shows dependence between AC peak current and pH of the supporting electrolyte. Amplitude: 40 mV, step potential: 4 mV, frequency: 50 Hz.

The acidity of the supporting electrolyte is one of the main factors that influence the morphology and height of the signals of electroactive compounds. Therefore, electrochemical behavior of acetaminophen on GFPE and MLGPE was initially studied in Britton–Robinson buffer (pH range from 2.0 to 9.0) by using the SWV technique. It was observed that the AC peak current was highly dependent on the pH of the supporting electrolyte (Figure 5) and that the AC signal deteriorates in the basic medium. For both GFPE and MLGPE, the oxidation signals of AC recorded by SWV were shifted positively with the decrease in pH and the plots of *E* vs. pH were linear with slope equal to 54.5 mV and 52.0 mV on GFPE and MLGPE, respectively. From the obtained *E*-pH relationship, it was concluded that an equal number of electrons and protons are involved in the AC oxidation process [47]. The mechanism of AC oxidation is well described in literature [48]—acetaminophen is electrochemically oxidized in a pH-dependent, 2-proton, 2-electron process to N-acetyl-*p*-quinoneimine (NAPQI). Depending on the pH, NAPQI undergoes various chemical transformations: hydrolysis in strong acidic media, hydroxylation in strong alkaline media and dimerization in intermediate pHs [61]. 

As the highest AC signals on both electrodes were observed in the acidic pH, other supporting electrolytes such as citrate and citrate-phosphate buffers were investigated. However, the best shape and height of the analyte signal were obtained in BR buffer. As shown in Figure 5, the peak currents of AC increased with the increase in pH, reaching a maximum at pH 5.0, and pH 4.0 at GFPE and MLGPE, respectively. Then peak current decreased with a further increase in the pH value of the solution. This indicates that the pH value of BR buffer affects the determination of AC. Therefore, BR buffer pH 5.0 and pH 4.0, using GFPE and MLGPE, respectively, were chosen as supporting electrolytes for subsequent measurements.

In the next step, the impact of the SW parameters (amplitude: 10–100 mV, frequency: 10–100 Hz, and step potential: 1–10 mV) on the AC signal was investigated. The results demonstrated a significant influence of the SW parameters on acetaminophen signals. In addition, the parameters of the SWSV technique, such as deposition potential and deposition time, were optimized. For step potential, the peak height increased up to 3 mV and then decreased for both electrodes. Then, the effect of frequency was studied. By using GFPE and MLGPE, a linear relationship was obtained between the peak current and frequency; however, at frequency values higher than 80 Hz and 50 Hz, the peak shape was distorted for GFPE and MLGPE, respectively. The AC signal also depended on the amplitude value. The acetaminophen peak current increased linearly to 30 and 80 mV for GFPE and MLGPE, respectively, and then remained constant. The influence of the deposition potential *E*_acc_ on the oxidation peak of AC was studied over the potential range of 0.0–0.5 V for GFPE and 0.0–0.6 V for MLGPE. The relationship between the stripping peak current and *E*_acc_ showed the maximum when deposition potential was 0.4 V. The influence of deposition time (*t*_acc_) was also investigated over the range from 0 to 180 s. Variation on the deposition time showed that the AC peak current increased initially with the deposition time up to 20 s, and then gradually leveled off. All further experiments were performed using these optimized parameters.

### 3.6. Analytical Characterization

**Figure 6:**
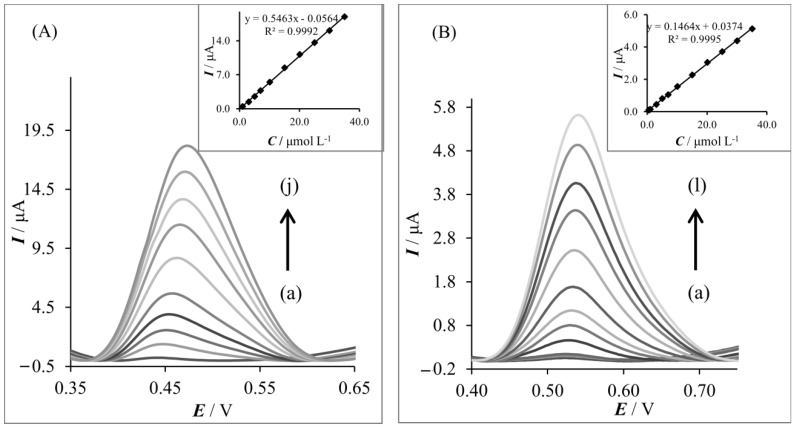
(**A**) SWS voltammograms of AC on GFPE: (a) 1.0 μmol L^−1^, (b) 3.0 μmol L^−1^, (c) 5.0 μmol L^−1^, (d) 7.0 μmol L^−1^, (e) 10.0 μmol L^−1^, (f) 15.0 μmol L^−1^, (g) 20.0 μmol L^−1^, (h) 25.0 μmol L^−1^, (i) 30.0 μmol L^−1^, and (j) 35.0 μmol L^−1^. The inset shows the corresponding calibration curve. (**B**) SWS voltammograms of AC determination on MLGPE: (a) 0.5 μmol L^−1^, (b) 0.7 μmol L^−1^, (c) 1.0 μmol L^−1^, (d) 3.0 μmol L^−1^, (e) 5.0 μmol L^−1^, (f) 7.0 μmol L^−1^, (g) 10.0 μmol L^−1^, (h) 15.0 μmol L^−1^, (i) 20.0 μmol L^−1^, (j) 25.0 μmol dm^−3^, (k) 30.0 μmol L^−1^, and (l) 35.0 μmol L^−1^. The inset shows the corresponding calibration curve.

The applicability of the proposed SWV and SWSV procedures for the determination of acetaminophen by employing both working electrodes was examined by measuring the AC peak current as a function of its concentration under the optimized conditions (n = 3). An SWV linear response was observed for the acetaminophen concentration range of 1.0–10.0 μmol L^−1^ and 3.0–30.0 μmol L^−1^ for GFPE and MLGPE, respectively. The SWSV response of AC at the GFPE and MLGPE increased linearly with the increase in acetaminophen concentration in the range of 1.0–35.0 μmol L^−1^ and 0.5–35.0 μmol L^−1^, respectively. Figure 6 shows the calibration graphs obtained by SWSV techniques for acetaminophen determination. The analytical characteristics of obtained calibration curves are presented in Table 1. From the obtained results, it can be concluded that the accumulation process provided a wider linear range. In Table 2, the experimental results of AC determination obtained on various CPEs were presented.

The analytical utility of the method was assessed by applying it to acetaminophen determination in pharmaceutical formulations. Acetaminophen determination was performed using the standard addition method for four commercially available pharmaceutical formulations: Apap, Apap Extra, Panadol, and Paracetamol Polfa Łódź. The composition and content of these drugs are described in Experimental section. The following experiments were only conducted with SWSV because of the better analytical performance of SWSV procedure. 

Samples of pharmaceutical formulations were subjected to three successive additions of the AC standard solution. SWS voltammograms were registered for the investigated sample and after each addition of the acetaminophen standard solution. The amount of determined compound in the pharmaceutical formulation is reported in Table 3. The recoveries of acetaminophen determination on MLGPE ranged from 98.3%–101.5%, while on GFPE, the recoveries were not much worse and ranged from 93.3%–103.6%. These data clearly demonstrate that the developed procedures can be successfully applied to AC determination in the pharmaceutical samples, especially taking into account that the European and national regulations stipulate a deviation of ± 5% of the declared content of the active substance in tablets [69]. 

## 4. Conclusions

In this paper, the electroanalytical performance of two CPEs was compared. The preparation of both CPEs, GFPE and MLGPE, is very simple, fast, and environmentally friendly. 

On the basis of Raman spectra, the differences in the structure of carbon materials were examined. It was revealed that bands of multilayer graphene are shifted in relation to the corresponding bands in graphite. The Raman investigations confirmed that the structure of both carbon materials was different, and therefore, various electrochemical properties can be expected for pastes made from these materials. 

Using scanning electron microscopy the topography and morphology characterization of MLGPE and GFPE were performed. MLGPE has an extremely smooth surface morphology with tightly distributed flakes. In contrast, the GFPE surface exhibits thin film with wrinkled structure.

Results of cyclic voltammetric studies indicate that GFPE has a larger electroactive surface area and better conductive properties than MLGPE. The calculated active area of the electrodes’ surfaces was equal to 5.14 ± 0.07 mm^2^ and 4.40 ± 0.06 mm^2^ for GFPE and MLGPE, respectively. The roughness factor for GFPE (RF = 0.741) and for MLGPE (0.622) was also calculated.

Both working electrodes were used for acetaminophen determination. Using the SWSV technique it can be seen that a wider linear range and lower LOD were obtained on MLGPE. While utilizing GFPE, higher AC signals and better sensitivity were achieved.

The developed analytical methods were successfully applied for acetaminophen determination in commercially available pharmaceutical formulations with good recovery. 

Collectively, both proposed paste electrodes are a promising tool for further electrochemical applications. However, GFPE can be more widely used due to its better electrochemical properties and the cheaper material from which it is made.

## Figures and Tables

**Table 1:** Comparison of AC determination parameters with SWV and SWSV on GFPE and MLGPE (n = 3).

Parameter	GFPE		MLGPE	
	SWV	SWSV	SWV	SWSV
Linear range [μmol L^−1^]	1.0–10.0	1.0–35.0	3.0–30.0	0.5–35.0
LOD [μmol L^−1^]	0.17	0.15	0.66	0.12
LOQ [μmol L^−1^]	0.57	0.49	2.20	0.39
R^2^	0.9995	0.9992	0.9988	0.9995

**Table 2:** Experimental results for various CPEs used for AC determination.

Electrode	Method	LOD [μmol L^−1^]	Linear Range [μmol L^−1^]	Ref.
CPE	LSV	–	3.0–7500	[39]
N-(3,4-dihydroxyphenetyl)-3,5-dinitrobenzamide-MWCNT modified CPE	DPV	10.0	15.0–270.0	[62]
Thionine immobilized MWCNT modified CPE	DPV	0.05	0.1–100.0	[63]
Pt nanoparticles-MWCNT modified CPE	DPV	0.7	0.4–60.0	[64]
Ethynylferrocene and NiO-MWCNT modified CPE	SWV	0.5	0.8–600.0	[65]
Graphene and CoFe_2_O_4_ nanoparticles modified CPE	SWV	0.025	0.03–12.0	[66]
Banana-hydrogel CPE	SWV	1.6	10.0–250.0	[67]
Zeolite modified CPE	CV	0.05	0.5–80.6	[68]
GFPE	SWSV	0.15	1.0–35.0	This work
MLGPE	SWSV	0.12	0.5–35.0	This work

**Table 3:** The results of AC determination in pharmaceutical formulations by using the standard addition method on GFPE and MLGPE (n = 3).

	GFPE			
Drug	Declared [mg]	Found [mg]	RSD [%]	Recovery [%]
Apap	500	466 ± 19	3.5	93.3
Apap extra	500	497 ± 19	3.4	99.4
Panadol	500	518 ± 6	1.1	103.6
Paracetamol Polfa Łódź	500	489 ± 16	2.8	97.8
	MLGPE			
Drug	Declared [mg]	Found [mg]	RSD [%]	Recovery [%]
Apap	500	507 ± 15	2.7	101.5
Apap extra	500	497 ± 8	1.4	99.3
Panadol	500	492 ± 4	0.7	98.3
Paracetamol Polfa Łódź	500	493 ± 16	2.9	98.5
